# Efficacy of ChatGPT in Educating Patients and Clinicians About Skin Toxicities Associated With Cancer Treatment

**DOI:** 10.2196/54919

**Published:** 2024-11-20

**Authors:** Annie Chang, Jade Young, Andrew Para, Angela Lamb, Nicholas Gulati

**Affiliations:** 1 Department of Dermatology, Icahn School of Medicine at Mount Sinai, 5 East 98th St, 5th Floor, New York, NY, 10029, United States, 1 212-241-9728

**Keywords:** artificial intelligence, ChatGPT, oncodermatology, cancer therapy, language learning model

## Abstract

This study investigates the application of ChatGPT, an artificial intelligence tool, in providing information on skin toxicities associated with cancer treatments, highlighting that while ChatGPT can serve as a valuable resource for clinicians, its use for patient education requires careful consideration due to the complex nature of the information provided.

## Introduction

Cancer therapy often results in systemic side effects that manifest as skin toxicities [1]. While oncologists regularly interact with patients undergoing treatment, they may not possess specialized dermatological knowledge. Similarly, dermatologists may lack insights into the nuances of cancer treatment–related skin conditions. This underscores the need for a collaborative approach, to manage these complications effectively and educate patients about them. Artificial intelligence (AI) tools such as ChatGPT can enhance this effort by providing comprehensive, accessible medical information [2,3]. This study evaluates ChatGPT’s effectiveness in offering detailed information on cancer treatment–related skin toxicities, aiming to bridge the gap between patient education and medical professionals’ expertise.

## Methods

### Overview

We developed 22 patient-oriented and 18 oncologist-oriented questions regarding the management of cancer treatment–related skin toxicities, based on our clinical experience and research on patients undergoing cancer therapy and designed to mirror common issues observed in clinical practice. Responses to these questions were generated using ChatGPT (OpenAI) version 3.5 (Supplementary Material S1 in Multimedia Appendix 1) [4].

Three board-certified dermatologists (AL, NG, AP) specializing in oncodermatology and affiliated with a tertiary academic institution in New York City evaluated these responses. Accuracy was assessed on a scale of 1 (completely inaccurate) to 5 (completely accurate), while comprehensiveness was rated on a scale of 1 (not at all comprehensive) to 5 (extremely comprehensive). The Flesch Reading Ease Score (FRES) was interpreted on a scale of 0 (extremely difficult to read, professional level) to 100 (extremely easy to read, fifth-grade level) and calculated using an online readability tool [5]. Interrater reliability was calculated to assess the consistency of ratings across reviewers.

### Ethical Considerations

This study did not involve human subjects or patient data and was therefore exempt from institutional review board approval.

## Results

Accuracy scores (out of 5) averaged 4.57 (SD 0.71) for patient questions and 4.54 (SD 0.68) for oncologist questions. Comprehensiveness scores (out of 5) averaged 4.43 (SD 0.69) for patient questions and 4.37 (SD 0.80) for oncologist questions. The average FRES scores were 41.9, 47.5, and 36.0 (overall, patient, and oncologist responses, respectively), all indicating college-level comprehension. Most (13/18, 72%) oncologist responses were unanimously deemed suitable for a patient-facing educational platform (Table 1). Interrater reliability analysis for all responses demonstrated a fair level of agreement between reviewers (27.7%; Fleiss κ coefficient of 0.227; *P*<.001) (Table 2).

**Table 1.** Twenty-two patient questions and 18 oncologist questions generated based on prior consultations received by the Dermatology Department at the Icahn School of Medicine at Mount Sinai from oncologists and graded on accuracy, comprehensiveness, and reading level.

Questions			Accuracy (for each of the 3 reviewers; 1-5)	Comprehensiveness (for each of the 3 reviewers; 1-5)	Flesch Reading Ease Score (0-100)
Patient questions					
	General questions				
		How will my skin change on chemotherapy?	5/5/4	4/5/4	49.7
		What types of chemotherapy cause my hair to fall out?	5/5/5	3/5/4	47.7
		What types of chemotherapy cause skin reactions?	5/5/5	3/4/3	42.5
		How often should my doctor monitor my skin during cancer treatment to stay on top of any changes?	4/3/5	4/3/5	41.3
		How long might skin reactions last after finishing my cancer treatment?	4/3/5	4/3/5	46.4
		Why am I seeing skin changes on immunotherapy treatment?	5/4/3	4/5/3	33.2
		How should I take care of my skin while on Keytruda treatment?	5/5/4	4/5/5	54.4
		After completing cancer treatment with Taxol, what long-term effects could there be on my skin?	5/5/5	4/5/5	42.3
	Evaluation questions				
		I am starting treatment with Taxol. Could you explain what skin side effects I should expect during this treatment?	5/5/4	5/5/4	50.5
		I am starting treatment with radiation therapy. Could you explain what skin side effects I should expect during this treatment?	5/5/5	4/5/5	47.7
		I am getting a bone marrow transplant. Could you explain what skin side effects I should expect during this treatment?	5/5/5	4/5/5	45.0
		I developed a rash on my face after starting Keytruda. What could be causing this?	5/4/3	4/5/4	36.3
		My nails have started separating from the nail bed after treatment with Tarceva. Is this normal and what should I do?	5/5/5	4/5/5	39.4
		I’m feeling depressed about the blisters on my feet from chemotherapy. Do you have any advice for coping, physically and mentally?	5/5/5	4/5/5	47.7
		I’m concerned about the changes I’ve noticed in my skin texture since starting Taxol. When should I contact my doctor regarding these changes?	5/5/4	4/5/5	55.9
	Management questions				
		Treatment with Tagrisso has caused me to have acne. How can I best manage this side effect?	4/5/5	4/5/5	56.4
		My skin has become very itchy since starting Keytruda. What should I do?	4/5/5	4/5/5	48.0
		I started getting blisters after radiation therapy. How can I best manage this side effect?	5/5/4	3/5/5	47.0
		Since starting methotrexate, I have started to lose a lot of hair. What should I do to prevent hair loss?	4/5/2	4/4/3	66.1
		I have very dry, cracked skin on my hands since taking Taxol. What moisturizers or creams can help with this?	4/5/2	4/4/3	66.1
		What types of moisturizers would you recommend for my skin during radiation therapy?	5/5/4	4/5/5	40.8
		My skin is more prone to sunburn since starting treatment with Xeloda. Are there specific sunscreen recommendations I should follow?	5/5/5	5/5/5	52.7
Oncologist questions					
	General questions				
		I am an oncologist. What preventive measures can I take to minimize the risk of skin reactions in patients undergoing radiation therapy?	5/5/4	3/5/5	46.1
		What topical treatments are recommended for skin reactions on chemotherapy treatment?	5/5/3	4/5/4	36.9
		What types of skin reactions are most commonly seen with Tagrisso?	5/5/4	4/5/5	48.5
		What distinguishes between mild, moderate, and severe skin reactions on immunotherapy treatment?	4/5/5	4/5/5	27.1
		Are there certain patients who may be more susceptible to severe skin reactions during cancer treatment?	4/5/5	4/5/5	39.2
	Evaluation questions				
		I am an oncologist treating a patient with Gleevec. What types of rashes warrant holding this therapy?	4/5/4	4/5/5	17.9
		I am an oncologist treating a patient with Taxol. What types of rashes warrant holding this therapy?	4/5/4	3/5/5	17.8
		I am an oncologist. If a rash resolves but then recurs for my patient on Herceptin, could it be a sign of allergy?	4/5/5	4/5/5	32.4
		I am an oncologist. My patient has formed blisters on the hand and feet since starting Xeloda. When should I consider a dermatology consult?	4/5/5	4/5/5	27.1
		I am an oncologist treating a patient with 5-FU^a^. What features help distinguish between rashes needing dermatology consult versus those I can manage?	4/5/5	5/5/5	33.1
	Management questions				
		I am an oncologist and my patient undergoing treatment with hydroxyurea is experiencing hair loss. How should I counsel this patient regarding hair regrowth?	5/5/5	4/5/5	49.0
		I am an oncologist. My patient is on Gleevec and experiencing blisters on their skin. How should I treat them?	4/5/3	5/5/3	44.0
		I am an oncologist and my patient is experiencing hand-foot syndrome after starting Xeloda treatment. What are the best approaches to manage this condition?	5/3/3	4/4/4	45.5
		I am an oncologist treating a patient with 5-FU. How can I guide them in caring for their nails to prevent discoloration and brittleness?	5/5/4	3/5/5	53.1
		I am an oncologist observing rashes and blisters in my patient taking Padcev. How should I treat them?	5/5/3	4/3/3	40.9
		I am an oncologist and my patient has a grade 2 maculopapular rash. Do I need to give systemic steroids for the rash?	5/5/5	3/5/5	32.8
		I am an oncologist and my patient has a grade 3 maculopapular rash. Do I need to give systemic steroids for the rash?	5/5/5	3/5/5	26.2
		I am an oncologist. When should I consider dose reductions for my patient on radiation therapy?	5/5/5	3/5/5	31.2

a 5-FU: 5-fluorouracil.

**Table 2.** Assessment of interrater reliability by question type.

Question type	Percent agreement	Fleiss κ coefficient	Fleiss κ coefficient *P* value	Strength of agreement
All responses				
All questions	27.7	0.227	<.001	Fair agreement
General questions	19.4	0.103	.10	Slight agreement
Evaluation questions	34.5	0.246	<.001	Fair agreement
Management questions	29.3	0.290	<.001	Fair agreement
Patient question responses				
All questions	20.5	–0.118	.08	Poor agreement
General questions	18.8	–0.130	.22	Poor agreement
Evaluation questions	28.6	–0.189	.18	Poor agreement
Management questions	14.3	–0.124	.30	Poor agreement
Oncologist question responses				
All questions	33.3	0.358	<.001	Fair agreement
General questions	20	0.243	<.001	Fair agreement
Evaluation questions	40	0.359	<.001	Fair agreement
Management questions	37	0.386	<.001	Fair agreement

## Discussion

Our findings suggest that ChatGPT holds promise as a resource for both patients and clinicians navigating the complexities of cancer treatment–related skin toxicities, given the relatively high levels of accuracy and comprehensiveness of its responses. However, the college reading level of ChatGPT’s responses poses a potential hurdle to widespread use; ChatGPT may currently be a more appropriate tool for clinicians, who will be able to comprehend its responses more uniformly compared to patients. It will likely be practically utilized by oncologists to complement their clinical judgment and that of dermatologists, particularly as AI-driven tools become increasingly integrated into clinical settings.

Reviewers identified occasional redundancies, irrelevant information, and minor inaccuracies in ChatGPT’s responses. They noted the need for its responses to be more evidence-based and to offer more up-to-date clinical recommendations when addressing oncologist questions. For instance, when responding to a question regarding the treatment of a patient experiencing rashes and blisters while taking enfortumab, ChatGPT did not recognize Stevens-Johnson syndrome as a potential concern. It only suggested “temporarily” holding the medication, even though current research recommends “permanently” discontinuing enfortumab in cases of suspected Stevens-Johnson syndrome [6]. These observations underscore that although ChatGPT generally provides useful information and could streamline its dissemination, its responses still require refinement, careful implementation, and regular monitoring to be considered for clinical use.

Integrating AI into dermatology-related patient education raises several technical and ethical considerations, including patient privacy, potential biases in AI responses, and the vital need to keep AI models current with the latest dermatology guidelines. A limitation of our study is the use of a single AI model; a comparison of ChatGPT with other models would provide a more rounded perspective on the capabilities of AI in this context.

Future research should involve incorporating additional metrics, such as clinical applicability and impact on patient outcomes, to provide a more comprehensive evaluation of ChatGPT’s potential in clinical settings. Studies with larger sample sizes, broader diversity of questions, and wider ranges of evaluators will improve our findings’ generalizability. It would be valuable to study how variations in prompt formulation affect the accuracy and comprehensiveness of ChatGPT’s responses. These enhancements would further improve ChatGPT’s ability to support both patient education and clinical decision-making in the context of cancer therapy–related skin toxicities.

## Supplementary material

10.2196/54919 Multimedia Appendix 1 Sample responses created by ChatGPT.

## Abbreviations

AI: artificial intelligence
FRES: Flesch Reading Ease Score
