# Supplementary material for: Efficacy of ChatGPT in Educating Patients and Clinicians About Skin Toxicities Associated With Cancer Treatment
Source: JMIR Dermatol. 2024 Nov 20;7:e54919. doi: 10.2196/54919 (PMC11600653; doi:10.2196/54919)
Supplement: Multimedia Appendix 1 [file derma-v7-e54919-s001.docx]

**Supplementary Material S1**

The following questions illustrate sample responses created by ChatGPT.

**Question 1 (Patient)**

Accuracy: 3/5/5

Comprehensiveness 4/5/5

Reading level: College-level

#### Q: My skin has become very itchy since starting Keytruda. What should I do?

A: Itchiness (pruritus) can be a bothersome side effect of Keytruda (pembrolizumab) treatment, which is an immunotherapy drug used to treat various types of cancer. It's important to address and manage itchiness to improve your comfort and overall well-being. Here are some steps you can take to alleviate itchiness caused by Keytruda:

1. Notify Your Healthcare Provider: First and foremost, inform your oncologist or healthcare provider about the itchiness you are experiencing. They can assess the severity of the issue, determine if it's related to Keytruda, and recommend appropriate management strategies.

2. Avoid Hot Water: Use lukewarm water when showering or bathing. Hot water can further dry out your skin and worsen itchiness.

3. Gentle Cleansing: Use a mild, fragrance-free soap or cleanser when washing your skin. Avoid harsh products that may irritate your skin further.

4. Moisturize: Apply a gentle, fragrance-free moisturizer regularly to keep your skin hydrated. Moisturizing can help alleviate dryness and itchiness.

5. Cool Compresses: Apply cool, damp cloths or compresses to the itchy areas. This can provide temporary relief from itchiness.

6. Topical Treatments: Your healthcare provider may recommend or prescribe topical treatments, such as creams or ointments, to soothe itchiness.

7. Antihistamines: Over-the-counter or prescription antihistamines can help reduce itchiness. Consult your healthcare provider before using any new medication.

8. Hydrate: Drink plenty of water to keep your skin hydrated from the inside out.

9. Loose Clothing: Wear loose-fitting, breathable clothing made of natural fibers to minimize irritation to your skin.

10. Avoid Scratching: Although it may be tempting, scratching can worsen itchiness and potentially lead to skin damage or infection. Try using a gentle patting motion instead of scratching.

11. Cotton Gloves: If you're experiencing itchiness on your hands, wearing soft cotton gloves at night can help prevent scratching while you sleep.

12. Humidifier: Consider using a humidifier in your living space to add moisture to the air, which can help alleviate dry skin and itchiness.

13. Mindfulness and Relaxation: Techniques such as deep breathing, meditation, and mindfulness can help reduce stress, which can contribute to itchiness.

It's important to work closely with your healthcare provider to address itchiness and any other side effects you may experience while on Keytruda. They can provide personalized guidance and recommendations based on your individual situation. If itchiness is severe, persistent, or affecting your quality of life, be sure to reach out to your healthcare provider promptly for assistance.

**Question 2 (Oncologist)**

Accuracy: 5/5/3

Comprehensiveness: 4/3/3

Reading level: College-level

#### Q: I am an oncologist observing rashes and blisters in my patient taking Padcev. How should I treat them?

Padcev (enfortumab vedotin) is an antibody-drug conjugate used to treat specific types of advanced urothelial cancer. Rashes and blisters can be potential skin side effects of Padcev treatment. If you're observing rashes and blisters in your patient, it's important to address these symptoms promptly. Here are steps you can take to treat them:

1. Assess the Severity:

- Evaluate the extent, location, and characteristics of the rashes and blisters. Determine if there are any signs of infection, such as redness, warmth, swelling, or pus.

2. Hygiene and Care:

- Advise the patient to keep the affected area clean and dry to prevent infection. Avoid picking or popping the blisters.

3. Topical Treatments:

- Apply over-the-counter antibiotic ointment to any open blisters to prevent infection. Consider using sterile gauze or non-stick bandages to cover the affected area.

4. Cool Compresses:

- Apply cool, damp cloths or compresses to the affected areas to help alleviate discomfort and reduce inflammation.

5. Avoid Irritants:

- Advise the patient to avoid using harsh soaps, fragrances, or other products that may irritate the affected skin.

6. Pain Relief:

- Over-the-counter pain relievers, such as acetaminophen or ibuprofen, may help manage discomfort. Ensure that the patient's overall health allows for the use of these medications.

7. Consult a Dermatologist:

- For severe or persistent cases of rashes and blisters, involve a dermatologist for specialized evaluation and treatment.

8. Medication Adjustment:

- Depending on the severity of the rashes and blisters and the patient's overall condition, consider discussing with the patient's oncologist the possibility of adjusting the dose of Padcev or temporarily holding the medication.

9. Supportive Care:

- Provide supportive care and recommendations for managing the symptoms, such as using gentle cleansers, avoiding friction, and keeping the skin well-moisturized.

10. Regular Follow-Up:

- Schedule regular follow-up appointments to monitor the progression of the rashes and blisters and adjust management strategies as needed.

11. Patient Education:

- Educate your patient about the potential skin side effects of Padcev and the importance of reporting any changes or worsening of symptoms.

Collaboration with a dermatologist and open communication with the patient are key to effectively managing skin-related side effects like rashes and blisters. By providing appropriate care and support, you can help your patient maintain their comfort and quality of life during their cancer treatment journey.
